# Diagnostic dilemmas in a girl with acute glomerulonephritis: Questions

**DOI:** 10.1007/s00467-017-3625-4

**Published:** 2017-03-20

**Authors:** Farah A. Falix, Michiel J. S. Oosterveld, Sandrine Florquin, Jaap W. Groothoff, Antonia H. M. Bouts

**Affiliations:** 1 Pediatric Nephrology of the Emma Children’s Hospital, Academic Medical Center, Amsterdam, Noord-Holland Netherlands; 2 Pathology of the Emma Children’s Hospital, Academic Medical Center, Amsterdam, Noord-Holland Netherlands

**Keywords:** Macroscopic hematuria, Acute kidney injury, Glomerulonephritis

## Case summary

A 6-year-old girl was referred to our unit with acute kidney injury. The week before, she was evaluated by her general practitioner for asymptomatic macroscopic hematuria. Three weeks earlier, she had complained of a sore throat accompanied by high fever for which her parents administered acetaminophen and ibuprofen. The general practitioner suspected her of having a urinary tract infection (UTI) and prescribed nitrofurantoin. A urinary culture was not obtained. Because of nausea and vomiting, nitrofurantoin was switched to amoxicillin/clavulanate. Four days after initiation of antibiotic treatment, macroscopic hematuria, nausea, and vomiting persisted. Therefore, she was referred to a regional hospital. Her previous medical history was unremarkable, and there were no other complaints. Her urinary output was possibly slightly decreased. Physical examination at the outpatient clinic was unremarkable (heart rate 93/min, respiratory rate 20/min, blood pressure 101/55 mmHg, temperature 36.7 °C). Laboratory investigations showed leukocytosis of 26.5 × 10E9/l, with normal hemoglobin and thrombocyte levels, disturbed renal function [creatinine 246 µmol/l (2.8 mg/dl); urea nitrogen 19.4 mmol/l (54 mg/dl)] with normal electrolytes and albumin level, elevated erythrocyte sedimentation rate (ESR) (68 mm/h), and C-reactive protein (CRP) (47 mg/l). Complement 3 and 4 levels were obtained, but results were not directly available. Urinalysis showed nephrotic-range proteinuria and hematuria. A renal ultrasound showed normal-sized, slightly hyperechogenic kidneys without signs of obstruction. Based on symptoms, laboratory results, and ultrasound findings, a diagnosis of acute kidney injury due to glomerulonephritis was made. The child was subsequently referred to our unit. On the day after admission, a Sunday, she became oliguric. Laboratory investigations revealed a further rise in creatinine to 524 µmol/l (5.9 mg/dl), electrolyte disturbances, nephrotic proteinuria, and hematuria (Table 1; laboratory results 1 day after admission).

**Table 1:** Laboratory results 1 day after admission

Tests	Results	Reference value
CRP	82 (H)	0–5 mg/l
Hemoglobin	6.2	6-9 mmol/l
Thrombocytes	498	150-600 10E9/l
Leukocytes	16.5	4–15 10E9/l
PT	11.2	9.7–11.9 s
aPTT	28	22–29 s
Sodium	131 (L)	135–145 mmol/l
Potassium	4.6	3.5-5 mmol/l
Chloride	89 (L)	98–107 mmol/l
Calcium	2.37	2.15–2.75 mmol/l
Phosphate	2.28 (H)	1–2.05 mmol/l
Creatinine	524 (H)	35–100 μmol/l
Urea nitrogen	28 (H)	1.8–6.4 mmol/l
Albumin	34	37–55 g/l
LDH	356	0-388 U/l
Complement C3	NA	0.9–1.8 g/l
Complement C4	NA	0.1–0.4 g/l
Urine		
Erythrocytes	>1000	0-17/ul
Protein/creatinine ratio	600	0–20 mg/mmol Cr

*PT* prothrombin time,* aPTT* activated partial thromboplastin time, *NA* not available, *L* low, *H* high,* Cr* creatinine

## Questions

1. What would be your differential diagnosis of the underlying cause of acute kidney injury?
2. What additional laboratory investigations would you perform?
3. Would you prepare the child for kidney biopsy at the first possible moment?
4. Would you blindly start immunosuppressive treatment in the weekend, without additional results?
5. How would you treat the child after renal biopsy and complement results?

